# Anticancer effects induced by artichoke extract in oral squamous carcinoma cell lines

**DOI:** 10.1186/s43046-020-00026-4

**Published:** 2020-04-05

**Authors:** Nadia Fathy Hassabou, Amina Fouad Farag

**Affiliations:** Oral and maxillofacial pathology, Faculty of Dentistry, October 6 University, Giza, 12585 Egypt

**Keywords:** Artichoke, SCC-25 cell lines, Cytotoxicity, Apoptosis

## Abstract

**Background:**

Oral squamous cell carcinoma is occupying the eighth position of all malignant neoplasia worldwide. Nowadays, natural compounds found in vegetables and fruits are important resources of many anticancer drugs especially those with high levels of phytochemicals representing an efficient strategy for cancer prevention and treatment. Artichoke (*Cynara cardunculus* L.) is a kind of antioxidant-rich vegetables demonstrated a potential anticancer activity on various types of cancer cells related to its content of phenolic compounds. Anticarcinogenic effects of polyphenolic extracts were reported to cause a reduction in cell viability, inhibition of cell growth, and initiation of apoptotic mechanisms. The present study aimed to investigate the cell cycle arrest, cytotoxic, and apoptotic effects of artichoke extract against the invasive oral squamous cell carcinoma.

**Results:**

A pure extract from the edible part and leaves of fresh artichoke was added to oral squamous carcinoma cell lines and to control group to evaluate the expression of caspase-9, Bcl-2, and Bax genes. Artichoke extract demonstrated the highest cytotoxic effect against cancer cell lines which increased in a time-dependent manner. No apparent effects were observed in the normal control group. Expression of Bax and caspase-9 genes revealed a highly significant increase in cancer cell lines (*p* = 0.0001) when compared to the control group. In addition to a highly significant decrease (*p* = 0.005) in Bcl-2 of cancer cells. It was demonstrated that artichoke extract induced cell growth arrest at G2/M phase which revealed a significant increase (*p* < 0.05) in comparison to the untreated control group.

**Conclusion:**

Artichoke exerts potent cell cycle arrest, cytotoxic, and apoptotic effects on oral squamous carcinoma cell lines.

**Graphical abstract:**

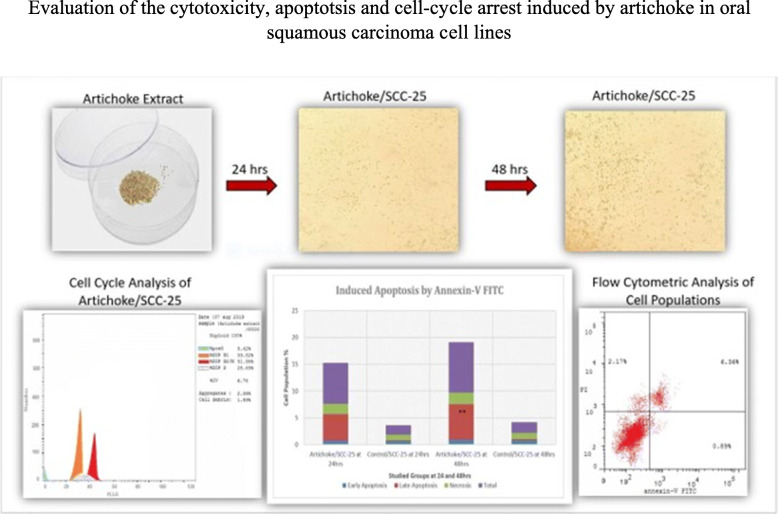

## Background

Oral cancer is considered one of the main causes of death worldwide with oral squamous cell carcinoma (OSCC) occupying the eighth position in the cancer incidence ranking, and its associated deaths reached 1310 or 0.25% of total deaths in Egypt in 2017 according to the latest WHO data. This aggressive epithelial malignancy is considered a threat to public health due to the capabilities of its cells to locally invade the surrounding tissues in addition to metastasis to distant sites which in turn is responsible for its poor clinical outcome and high morbidity rate [1, 2].

Despite advances in the early cancer detection and its therapeutic interventions, most malignancies are still diagnosed and treated at very advanced stages with poor overall survival and significant mortality rates probably due to the limited range of therapeutic modalities which classically consist of surgery, radiotherapy, and/or chemotherapy. Thus, new therapeutic approaches have been investigated and the most promising one is naturally acquired agents with known anticarcinogenic effects [3–5].

Many epidemiological studies over the past years suggest that diets particularly rich in vegetables and fruits have anticarcinogenic effects and apoptosis triggering properties [6–8]. These have been attributed to the presence of high levels of phytochemicals in vegetables and fruits which can be an efficient strategy for cancer prevention and treatment [9].

Nevertheless, the mechanisms of modulation and induction of apoptosis by these natural compounds are still unclear. Natural phytochemicals containing phenolic compounds have antitumor and anti-metastatic properties [10] that can be attributed at least in part to polyphenols both in vitro and in animal models [11].

The current growing interest in dietary plants has led to renewed interest in artichoke because of its high polyphenolic content. Artichoke polyphenols represent up to 2% of the fresh weight, mainly hydroxycinnamic derivates (0.5–1.5%), and in the edible part, they are mainly represented by mono- and di-caffeoylquinic acids. The bioavailability of metabolites of hydroxycinnamic acids after ingestion of cooked edible artichokes has also been demonstrated [12].

The flowers of artichoke (*Cynara cardunculus* L.) have a well-recognized history of consumption as a food, especially as part of the Mediterranean diet. This plant is usually cultivated for its leaves which are blanched and eaten as a vegetable (cardoon, artichoke). The edible, fleshy leaves (bracts) of the artichoke are cooked and eaten worldwide. They are also recognized for their potential therapeutic effects including inhibiting the biosynthesis of cholesterol, low-density lipoprotein oxidizing, mobilizing energy reserves, and inducing choleresis, along with antibacterial, antioxidant, and hepatoprotective effects [13]. These beneficial effects appear to be related to its content of phenolic compounds.

Flavones with their glycosides, caffeoylquinic acids, and triterpenoid saponins are active constituents found in the extract of *Cynara cardunculus* L. with several antioxidant and antigenotoxic activities [14].

Artichoke as an ancient plant grown mainly in Egypt, Italy, and Spain has been used for so long as a vegetable in traditional medicine against liver complaints. It showed marked choleretic, hepatoprotective, hypocholesterolemic, and antioxidative potentials. Furthermore, they exhibited cancer chemopreventive properties by triggering apoptosis on human hepatoma cells [15]. Mileo et al. [16] studied the anticarcinogenic effects of polyphenolic extracts from the edible part of artichokes. They observed a reduction in cell viability, inhibition of cell growth, and initiation of apoptotic mechanisms against the invasive breast cancer cell line with no effect on normal breast epithelial cell line. They reported chlorogenic acid (ChA) to be the most representative component of the polyphenolic fraction of artichoke as it triggered the apoptosis via a mitochondrial and a death-receptor pathway, as shown by the activation of caspase-9 (CASP-9) and CASP-8. Furthermore, an increase of the Bax-Bcl-2 ratio, upregulation of cyclin-dependent kinase inhibitor p21, WAF1, and a loss of mitochondrial transmembrane potential were documented.

The potential anticarcinogenic effects of artichoke extract on model cell cultures have not been thoroughly investigated and furthermore have never been studied on the oral cancer. Therefore, this study was conducted to examine the induction of cell cycle arrest, antiproliferative, and apoptotic effects of artichoke extract on the squamous cell carcinoma (SCC) cell lines.

## Methods

### Preparation of plant extract

The samples of the fresh artichoke (*Cynara scolymus* L.) were collected from Nahia region, Giza, Egypt, in February 2019. The cultivar is known as Balady. The formal identification of the samples was undertaken by authors, and a voucher specimen (numbered 5.2.2019) has been located at the herbarium associated with our institution. The edible part (head) and leaves of fresh artichoke were used for extract preparation. They were dried and ground into fine powder using a blender. To prepare the aqueous artichoke extract, each 10 g of powder was soaked within 200 ml boiling distilled water for 15 min and allowed to cool down at room temperature. To obtain ethanolic artichoke extract, 50 g of the ground herbs was soaked in 500 ml 50% ethanol solution for 24 h at room temperature, then the mixtures were filtered and passed sequentially through a 0.22-μm filter. The extract was concentrated by rotary evaporator and stored at – 20 °C at the National Research Institute, Egypt. The concentrated extracts were dissolved in dimethyl sulphoxide (DMSO) (Sigma, USA) to get a stock solution of 10 mg/mL [17].

### Cell culture protocol

Human lung normal cell lines (WI-38) and human tongue SCC cell lines (SCC-25) were obtained from American Type Culture Collection, and cells were cultured using DMEM (Invitrogen/Life Technologies) by the Holding Company for Biological Products & Vaccines, Egypt (VACSERA). Cells were maintained in RPMI 1640 culture medium (Gibco, USA) and supplemented with 10% FBS (Hyclone), 10 ug/ml of insulin (Sigma), and 1% antibiotic-antimycotic solution (penicillin-streptomycin) (Invitrogen, USA). All of the other chemicals and reagents were from Sigma and Invitrogen.

The culture medium was removed to a centrifuge tube. The cell layer briefly rinsed with 0.25% (w/v) Trypsin 0.53 mM EDTA solution to remove all traces of serum which contains Trypsin inhibitor. About 2.0 to 3.0 ml of Trypsin EDTA solution was added to the flask and the cells were observed under an inverted microscope until the cell layer is dispersed (usually within 5 to 15 min). To avoid clumping, do not agitate the cells by hitting or shaking the flask while waiting for the cells to detach. Cells that are difficult to detach may be placed at 37 °C to facilitate dispersal.

Complete growth medium (6.0 to 8.0 ml) was added, and then the cells were aspirated by gently pipetting. The cell suspension was transferred to the centrifuge tube with the medium and cells from the previous step and centrifuge at approximately 125×*g* for 5 to 10 min to discard the supernatant. Fresh growth medium was used to re-suspend the cell pellet, and appropriate aliquots of the cell suspension were added to new culture vessels. All cell culture experiments were carried out at 37 °C in a 100% humidified incubator containing 5% CO_2_.

Artichoke extract was added on the first day of treatment to SCC-25 cell lines and to healthy cell lines that have been used as a control. Incubation was carried out for 24 and 48 h at 37 °C, after the plates were examined under the inverted microscope and proceeded for the MTT assay. PCR was done after incubation. DNA and RNA were extracted from cells using quantitative real-time PCR.

### MTT–cytotoxicity assay protocol

Plate cells (cells density 1.2–1.8 × 10,000 cells/well) in a volume of 100 μl complete growth medium + 100 ul of the tested compound per well for 24 h before the micro-culture tetrazolium assay (MTT) assay.

MTT method of monitoring in vitro cytotoxicity is well suited for use with multi-well plates. Cultures were removed from the incubator into a laminar flow hood or other sterile work areas. Each vial of MTT [M-5655] was reconstituted to be used with 3 ml of medium or balanced salt solution without phenol red and serum.

Reconstituted MTT was added in an amount equal to 10% of the culture medium volume. Cultures were returned to the incubator for 2–4 h depending on cell type and maximum cell density. An incubation period of 2 h is generally adequate but may be lengthened for low cell densities or cells with lower metabolic activity.

After the incubation period, cultures were removed from the incubator and the resulting formazan crystals were dissolved by adding an amount of MTT solubilization solution [M-8910] equal to the original culture medium volume. Gentle mixing in a gyratory shaker was performed to enhance dissolution and spectrophotometrically measure absorbance at a wavelength of 570 nm. The background absorbance of multi-well plates was measured at 690 nm and subtracted from the 450-nm measurement.

### Annexin-V/PI dual staining assay

Fluorescein isothiocyanate (FITC) was used to perform a quantitative assessment of apoptosis by using propidium iodide (PI) flow cytometry (ab139418) detection kit/BD. Cells were cultured overnight (1.5 × 105 cells) in 25 cm^2^ cell culture flasks before addition of artichoke extract. Cells were then treated with IC50 value of the extract which is (102.74 ug/ml) for 24 and (184.81 ug/ml) for 48 h.

The positive control was prepared by culturing the control SCC-25 cell lines in medium containing 200 ml H_2_O_2_ for 30 min. Cells were collected, washed twice with cold PBS, and re-suspended in binding buffer (1 × 105 cells/ml). Cells were transferred to a tube with addition of 5 μl of FITC-conjugated annexin-V (annexin-V FITC) and 5 μl of PI (PI 50 mg/ml) followed by incubation for 15 min at room temperature.

The stained cells were diluted by the binding buffer and analyzed by the flow cytometer (BD FACSCalibur). Apoptotic cells are reflected by quantification of annexin-V FITC binding to externalized PS. In flow cytometry analysis, annexin-V/PI staining is based on the ability of the protein annexin-V to bind to PS, which is externalized in the outer cell membrane upon induction of apoptosis. Four different populations of cells were distinguished: cells that were unlabeled (*viable cells*), those that have bound to annexin-V FITC only (*early apoptotic*), those that have been stained with PI (*necrotic*), and those that have both bound to annexin-V FITC and been labeled with PI (*late apoptotic*).

The fluorescence distribution was displayed as a color dot plot analysis, and the fluorescent cell percentage in each quadrant was determined.

### Light microscopic studies

Inverted light microscope was used at magnification (×200) to investigate the morphological alterations of apoptosis induced by artichoke extract in both healthy and SCC-25 cell lines at 24 h and 48 h.

### Cell cycle analysis

Flow cytometry analysis was used to evaluate the percentage of DNA content in each phase of the cell cycle. SCC-25 cells were cultured in multi-well plates overnight and then treated with artichoke extract. The Cycletest^TM^ Plus DNA Reagent Kit (BioVision, USA) was used to determine the cell cycle phase distributions. Nuclei were isolated, stained with PI, and analyzed on FACSCalibur flow cytometer according to the instructions of the manufacturer. The percentages of DNA in each cell cycle phase were analyzed using ModFit software (ModFit, Topsham, ME, UK).

### Quantitative real-time PCR

#### RNA extraction

Total RNA was isolated from cell culture according to instructions of manufacture using Qiagen extraction kit (Qiagen, USA). RNA extraction was performed on in vitro cells. Centrifugation of SCC-25 cells was performed for 3 min at full speed. The supernatant was removed and transferred to a new micro-centrifuge tube. One volume of 70% ethanol (300 μl) was added to the cleared lysate.

About 700 μl of the sample was transferred to RNeasy spin column that was placed in a collection tube and centrifuged for 15 s at ≥ 8000 rpm. RNase-free water was added directly to the spin column membrane and centrifuged for 1 min at ≥ 8000 rpm to isolate the RNA.

The isolated RNA was transferred to a new Eppendorf tube and stored at – 80 °C. Quantification of RNA was performed in duplicate using spectrophometry at 260 nm (dual wavelength Beckman, Spectrophotometer, USA).

#### Sequence of real-time PCR primers

Primer sequences (5′ to 3′) for all studied genes are demonstrated (Table 1).

**Table 1:** Primers sequence for all studied genes in the work

Gene	Primer type	Primer sequences (5′ to 3′)
CASP-9	RT-PCR Forward/reverse	CASP9-F 5′-CCA GAG ATT GCG AAA CCA GAG G-3′ CASP9-R 5′-GAG CAC CGA CAT CAC CAA ATT C-3′
BAX	RT-PCR Forward/reverse	Bax F 5′-GCG AGT GTC TCA AGC GCA TC-3′ Bax R 5′-CCA GTT GAA GTT GCC GTC AGA A-3′
BCL-2	RT-PCR Forward/reverse	Bcl-2 F 5′-GATGTGATGCCTCTGCGAAG-3′ Bcl-2 R 5′-CATGCTGATGTCTCTGGAATCT-3′
β-actin	RT-PCR Forward/reverse	β-actin F 5′-TGC CGA CAG GAT GCA GAA G-3′ β-actin R 5′-GCC GAT CCA CAC GGA GTA CT-3′

#### cDNA synthesis

The total RNA (0.5–2 μg) was used for cDNA conversion using high-capacity cDNA reverse transcription kit (Fermentas, USA).

Moloney murine leukemia virus (MMLV) reverse transcriptase was used for the synthesis of cDNA from RNA. It is an RNA-dependent DNA polymerase that uses single-stranded RNA as a template in the presence of a primer to synthesize a complementary DNA strand.

Human placental ribonuclease inhibitor (HPRI) was used for inhibition of RNase activity. The cDNA master mix was prepared according to the kit instructions and was added for each sample. The mixture was incubated in the programmed thermal cycler for 1 h at 37 °C followed by inactivation of enzymes at 95 °C for 10 min and finally cooled at 4 °C. Then, RNA was changed into cDNA and the converted cDNA was stored at – 20 °C.

#### Real-time qPCR amplification

Real-time qPCR amplification and analysis were performed using an Applied Biosystem with software version 3.1 (StepOne™, USA). The qPCR assay with the primer sets was optimized at the annealing temperature. All cDNA was duplicated and including previously prepared samples. The thermal cycling conditions for RT-PCR comprised an initial denaturation step at 95 °C for 30 s, then 45 cycles at an appropriate annealing temperature depending on the primer set for 1 min.

#### Calculation of relative quantification (RQ)

Threshold cycle (Ct) number is the value where the PCR curve crosses the threshold in the linear part of the curve. Endogenous controls are the gene that does not vary between all of the samples tested. The calibrator is the sample that all others are compared to. It is the “untreated” or “time zero.” The RQ is a technique used to analyze the fold changes in gene expression in a given sample compared to a calibrator. These values were necessary for the calculations described below [18].

∆ Ct = Ct gene test – Ct endogenous control

∆∆Ct = ∆Ct sample – ∆Ct calibrator

RQ = Relative quantification = 2^−∆∆Ct^

## Statistical analysis

Scores of overall expressed genes were reported as mean values and standard deviation using SPSS (Statistical Package for Social Sciences) 16.0 software. Mean values between the studied groups were compared using paired samples Student’s *t* test. *P* value was considered highly significant when ≤ 0.01and significant when ≤ 0.05.

## Results

### MTT cytotoxicity assay

In the present work, the cytotoxic effect of artichoke extract was evaluated by MTT assay. It was observed that there was no apparent cytotoxicity of the artichoke extract on the healthy cell lines that have been used as a control group. The current results showed cytotoxic and antiproliferative effects of artichoke extract on SCC-25 cell lines which is in time-dependent manner that was represented as mean ± SD and showed a significant increase (*p* ≤ 0.05) from 24 to 48 h of influence (Table 2).

**Table 2:** Mean values and variability of measures (SD) of cytotoxicity of artichoke extract on SCC-25 cell lines at 24 and 48 h using paired samples Student’s *t* test

Sample code	Cytotoxicity IC50 ug/ml at 24 h	Cytotoxicity IC50 ug/ml at 48 h
	SCC-25	SCC-25
Artichoke extract/SCC-25	102.74 ± 7.91	184.81 ± 9.52

### Fold changes in gene expression

**Fig. 1:**
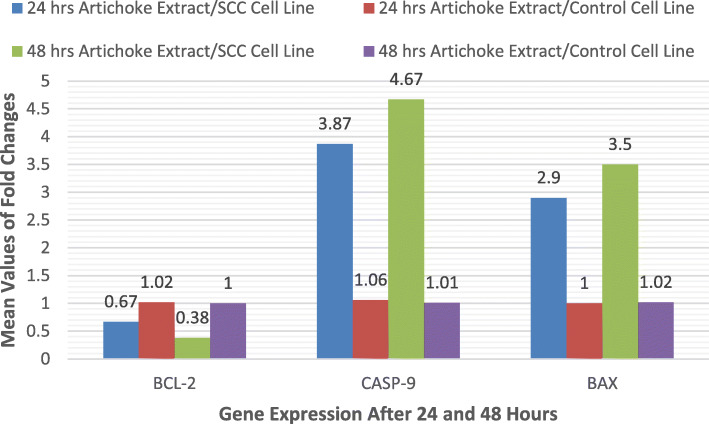
Mean values of fold changes in gene expression occurred in studied control and SCC-25 cell lines after 24 and 48 h

Cytotoxic and anti-proliferative effects of the artichoke were represented as mean ± SE values of gene expression (Table 3). A highly significant decrease was observed in Bcl-2 gene expression (*p* = 0.005). Expression of CASP-9 and BAX genes revealed a highly significant increase (*p* = 0.0001) when SCC-25 cell lines treated with the artichoke extract group compared to the control one (Fig. 1).

**Table 3:** Mean values and measures of variability (SE) of studied genes expression in control and artichoke SCC-25 cell lines in relation to the time of influence using paired Student’s t test

Sample code	Fold changes					
	BCL-2		CASP-9		BAX	
	24 h	48 h	24 h	48 h	24 h	48 h
Control/SCC-25	1.02 ± 0.02	1 ± 0.009	1.06 ± 0.03	1.01 ± 0.02	1 ± 0.07	1.020 ± 0.013
Artichoke/SCC-25	0.6734 ± 0.028	0.3800 ± 0.008	3.8570 ± 0.05	4.6367 ± 0.07	2.9034 ± 0.06	3.5766 ± 0.04

### Artichoke extract induced apoptosis in SCC-25

**Fig. 2:**
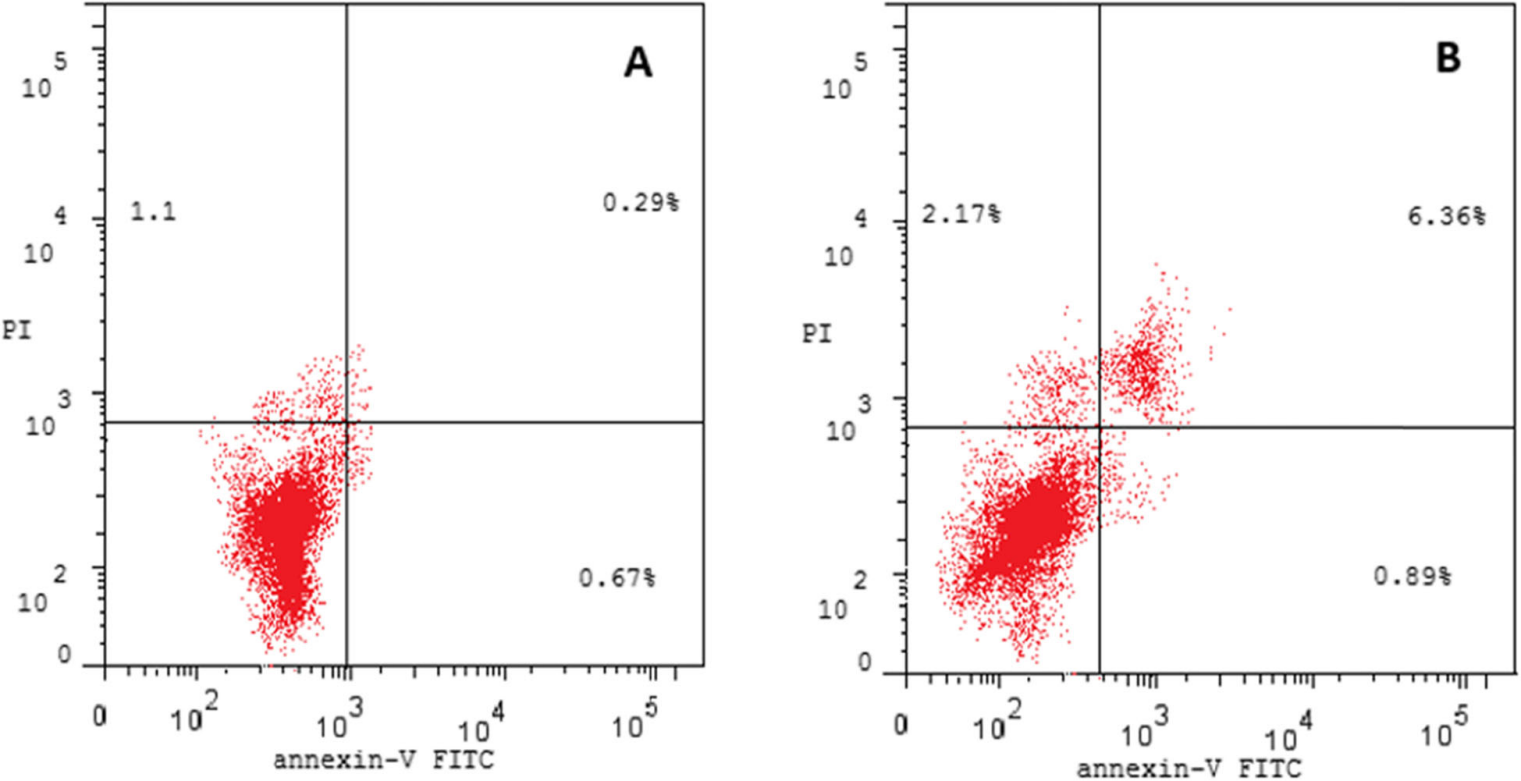
Annexin V/PI double-staining assay of control SCC-25 (**a**) and SCC-25 cell lines treated with artichoke extract at 48 h. The *Y*-axis represents the PI-labeled population, whereas the *X*-axis represents the labeled annexin-V FITC-positive cells. The lower left portion of the fluoro-cytogram (An-, PI-) shows viable cells, whereas the lower right portion (An+, PI-) shows early apoptotic cells. The upper right portion (An+, PI+) shows late apoptotic cells, while the upper left portion (An-/PI+) demonstrates the percentage of necrotic cells

In the current study, cell death induced by artichoke extract was investigated for apoptotic activity by monitoring phosphatidylserine (PS) translocation using the annexin-V FITC/PI assay. Four populations of cells were distinguished from our current results by Annexin-V/PI staining, viable cells (AnnV^−^/PI^−^), early apoptotic cells (AnnV^+^/PI^−^), late apoptotic cells (AnnV^+^/PI^+^), and necrotic cells (AnnV^-^/PI^+^) (Fig. 2a, b).

**Fig. 3:**
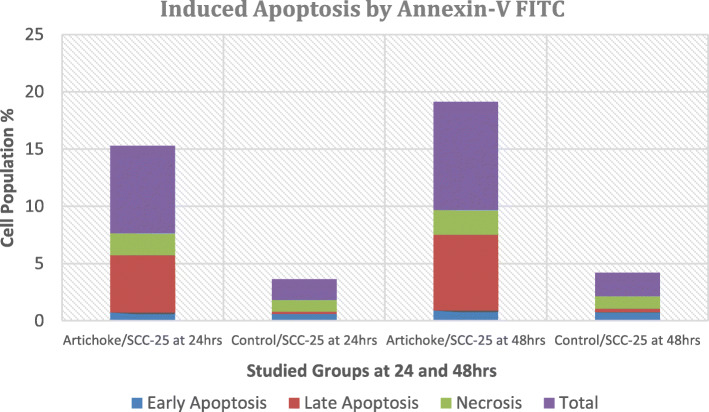
Percentage of cell populations by annexin-V FITC flow cytometry analysis in both control- and artichoke-treated SCC-25 that demonstrate a highly significant increase (*p* ≤ 0.001) in the late apoptosis of artichoke/SCC at 48 h as compared to the control group

The flow cytometry analysis of SCC-25 cell lines showed that through the treatment with artichoke extract, the cell population tended to shift from viable to apoptotic which is time dependent from 24 to 48 h. The early apoptotic rates were (0.75 and 0.89%), respectively demonstrating non-significant differences when compared with the rate of positive control (0.67%) (Table 4, Fig. 3).

**Table 4:** Percentages of distinguished cell populations by annexin-V FITC flow cytometry analysis in both control- and artichoke-treated SCC-25 cell lines using paired Student’s t test and the results were represented as mean ± SE

Sample data	Apoptosis				Necrosis		Total	
	Early		Late					
	24 h	48 h	24 h	48 h	24 h	48 h	24 h	48 h
Artichoke/SCC25	0.75 ± 0.05	0.89 ± 0.04	4.97 ± 0.19	6.36 ± 0.21	1.93 ± 0.03	2.17 ± 0.04	7.65 ± 0.21	9.42 ± 0.28
Control/SCC25	0.60 ± 0.02	0.67 ± 0.02	0.21 ± 0.03	0.29 ± 0.03	1 ± 0.02	1.1 ± 0.03	1.81 ± 0.04	2.06 ± 0.07

Furthermore, highly significant changes (*p* ≤ 0.001**) in the percentage of the late-stage apoptotic cells for artichoke extract-treated SCC-25 group were observed at 24 and 48 h of incubation (4.97 and 6.32%), respectively as compared to 0.29% of the positive control group (Table 4, Fig. 3).

Our study revealed that rates of necrotic cells have no significant differences when comparing positive control group to artichoke/SCC-25 group that were 1.1% and 2.17% respectively at 48 h. These results demonstrated the ability of artichoke extract to exert an apoptotic effect on SCC-25 cell lines particularly in the late stage of apoptosis which is an irreversible process.

### Light microscopic studies

Morphological investigation of apoptosis by inverted light microscope in our study revealed that artichoke extract induced cell death in SCC-25 cell lines by apoptosis, and no morphological changes were observed in normal healthy cell lines. Phenotypically, apoptosis is characterized by cell shrinkage, chromatin condensation, DNA fragmentation, and collapse of the cell into minute fragments (apoptotic bodies).

**Fig. 4:**
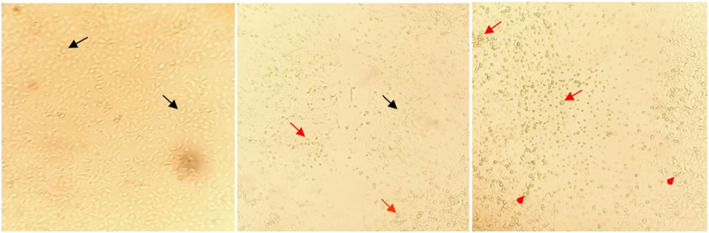
Morphological changes of SCC-25 cells treated with artichoke extract for **b** 24 h and **c** 48 h viewed under an inverted light microscope. Control SCC-25 (**a**) was also included (× 200 magnification). Black arrows showed normal viable cells while red arrows showed cell shrinkage and nuclear condensation due to apoptosis. Red arrowheads showed the presence of apoptotic bodies

Our microscopic results showed an increase in the number of apoptotic cells corresponding to the time of incubation with artichoke extract (Fig. 4). Round or oval masses of cytoplasm appeared which referred to apoptotic bodies.

### Cell cycle analysis

**Fig. 5:**
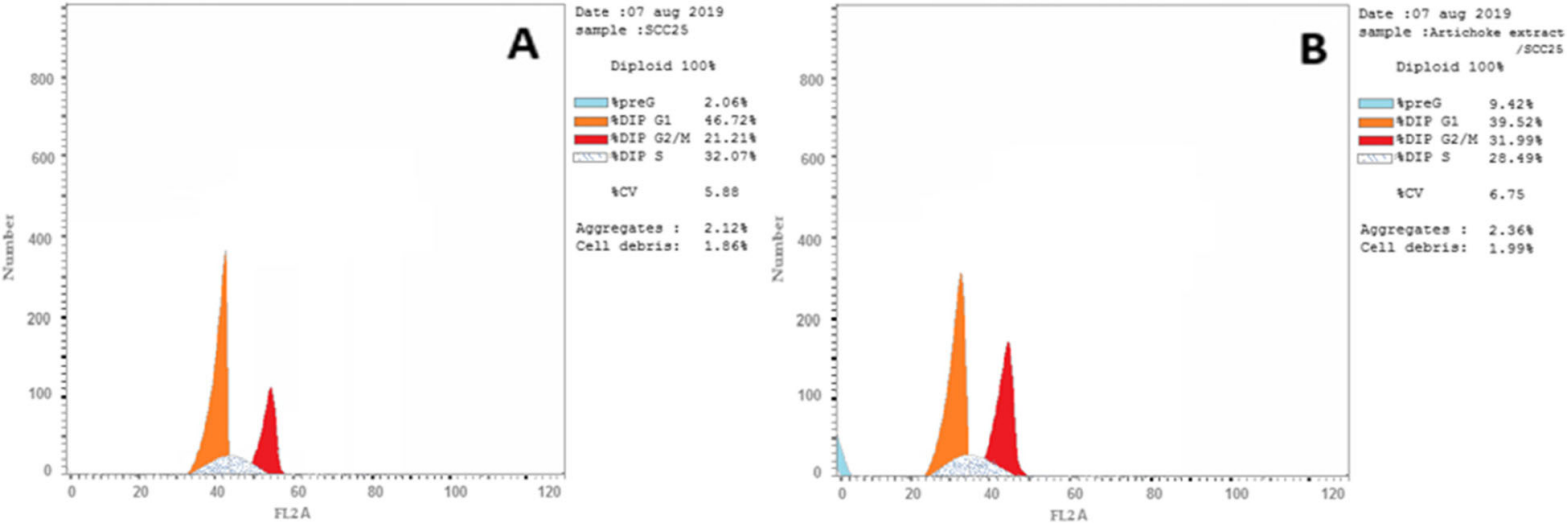
Artichoke extract induces cell cycle arrest in SCC-25 cell lines. Flow cytometric analysis was performed for cell cycle distribution. The DNA content was evaluated with PI staining, fluorescence measured, and analyzed. Representative flow cytometry graph for untreated control SCC-25 group (**a**) and artichoke-treated SCC-25 group (**b**)

**Fig. 6:**
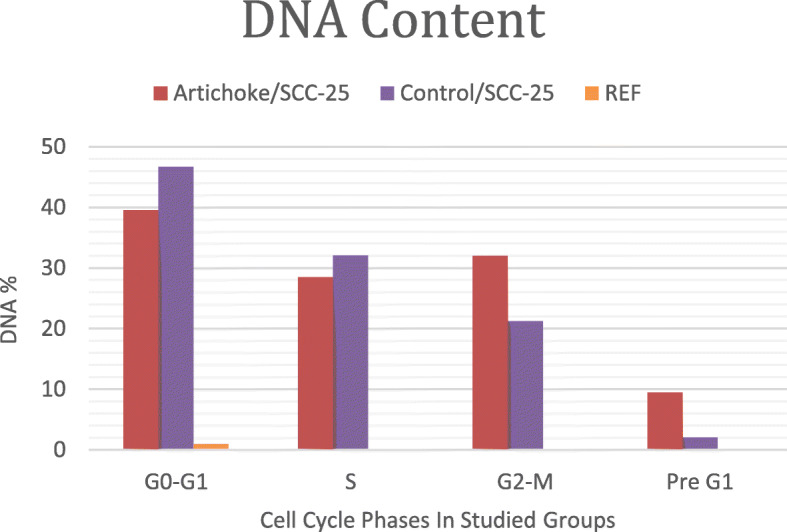
Percentage of DNA content in control and artichoke SCC cell lines during flow cytometry cell cycle analysis

After SCC-25 cells were incubated with artichoke extract, they were isolated to examine the effects on cell cycle by flow cytometry. The results of the current work demonstrated that artichoke extract induced cell growth arrest at G2/M phase (Fig. 5a, b). After 48 h of treatment with the artichoke extract, a significant increase (*p* < 0.05) in the number of cells arrested at the G2/M growth phase was observed as compared the control group of SCC-25, (Table 5, Fig. 6).

**Table 5:** Percentage of DNA content during phases of the cell cycle in control and artichoke extract/SCC25 using paired Student’s t test and variability of measures (SD)

Sample data	Results				
	DNA content %				
Sample code	G0-G1%	S%	G2-M%	Pre G1%	Comment
Artichoke extract/SCC25	39.52 ± 1.47	28.49 ± 1.82	31.99 ± 1.29	9.42 ± 0.22	Cell growth arrest at G2/M
Control/SCC25	46.72 ± 2.15	32.07 ± 1.29	21.21 ± 1.61	2.06 ± 0.09	–

## Discussion

Currently, cancer is one of the deadliest diseased worldwide. World Health Organization (WHO) in 2012 reported that 8.2 million people died with cancer. About 30% of the most common cancers such as breast, colorectal, cervical, and oral cancer can be prevented or curable if treated promptly [19].

Cancer is a disease characterized by unlimited proliferation of cells which may spread to different parts of the body. Angiogenesis is an important factor for proliferating and spreading of cancer cells. The whole process of tumorigenesis and rate of tumor progression depends on the balance between proliferation and apoptosis of the cancer cells [20].

Oxidative stress is one of the most important factors that cause cancer and a well-studied event that gives rise to the conditions leading to tumor onset and progression [4]. It has been demonstrated that continuous inflammation may lead to a preneoplastic situation. Chronically inflamed cells secrete a higher amount of reactive oxygen/nitrogen species which recruit more activated immune cells to overcome endogenous antioxidant response leading to the amplification of dysregulated processes and an irreversible oxidative damage to nucleic acids, proteins, and lipids which may cause genetic and epigenetic modifications. This leads to the dysregulation of oncogenes and tumor suppressor genes that drive the initiation of carcinogenesis [21].

Nowadays, it is well known that chemoprevention is a promising strategy which depends on natural dietary products and synthetic substances that not only offer protection against oxidative reaction but also provide important preventive mechanisms including suppression of cell proliferation, apoptosis, and modulation of epigenetic processes [22].

There are lines of evidence and many epidemiological studies indicating that medicinal plants and diets particularly rich in fruits and vegetables constitute a common alternative for cancer prevention and treatment in many countries around the world [23].

Many researchers worldwide demonstrated that anticancer beneficial effect of diets is attributable to polyphenol, brassinosteroid, and taxol compounds which have antiproliferative activities both in animal models and in humans [24]. Therefore, the current work was conducted to examine anticancer properties of artichoke extract on oral squamous carcinoma cell lines.

The artichoke is an ancient herbaceous plant, originating from the Mediterranean area. Today, it is widely cultivated all over the world [25]. It is a promising medicinal plant as its extracts exert antiproliferative, anti-invasive, and antimetastatic effects; induce apoptosis through modulation of antiapototic proteins such as Bcl-2; and increase p53, p21, and p27. Several antioxidant bioactive compounds had been identified in globe artichoke, and their antioxidant activities were confirmed in multiple pre-clinical studies [23].

The results of the current work confirmed that treatment with artichoke extract inhibits proliferation of cancer cell. The present results showed cytotoxic and antiproliferative effects of applied artichoke extract on cancer cells achieved from SCC-25 cell lines that represent significant increase which is time dependent. Also, no effects on the normal cells have been revealed. The obtained findings were in accordance with earlier studies that were conducted to evaluate antitumor and apoptotic properties of artichoke extract in different cancer cell lines [17, 22].

Apoptosis is a genetically programmed mechanism which promotes the beginning of tumorgenesis when subjected to inhibition. It is associated with the expression of two synergistically acting genes that encode anti- and pro-apoptotic proteins. It is well known that Bax gene promotes apoptosis while Bcl-2 gene enhances cell survival [26].

One of the most important events in carcinogenesis is the mutation of p53 tumor suppressor gene. Cells with a mutated p53 gene tend to escape from apoptosis. Apart from the cell-cycle regulation, increasing evidence suggests that caspase family proteases that play an important role in the mechanism of apoptosis. In apoptosis, caspase are indispensable enzymes that control the pathway [27].

Apoptotic frequency and antiproliferative potential in our study represented by an increase in the expression of CASP-9 and Bax genes which were inversely proportional to the decreased expression of Bcl-2 gene. These findings were in the same line of our previous study and were consistent with other studies [28, 29].

In our study, apoptotic cells from artichoke extract treatment showed that the cell cycle was arrested at G2/M phase. Moreover, defects in the G2/M arrest checkpoint allow a damaged cell to enter mitosis and undergo apoptosis and this enhances the increase of the cytotoxic effects of the therapeutic drugs [20, 29]. For this reason, our results suggest that artichoke extract induced cell-cycle G2/M phase arrest and apoptosis in SCC-25.

## Conclusion

Artichoke medicinal plants have the potential to become a safe anti-carcinogenic agent. It has a cytotoxic and inhibitory effect on cancer cell proliferation through induction of apoptosis. Further studies are needed to prove the anticancer activities of artichoke especially in head and neck cancers.

## Data Availability

Not applicable.

## Abbreviations

CASP-9: Caspase-9
ChA: Chlorogenic acid
Ct: Threshold cycle
FITC: Fluorescein isothiocyanate
HPRI: Human placental ribonuclease inhibitor
MMLV: Moloney murine leukemia virus
MTT assay: Micro-culture tetrazolium assay
OSCC: Oral squamous cell carcinoma
PI: Propidium iodide
PS: Phosphatidylserine
SCC: Squamous cell carcinoma
